# Comparison of different surgical treatments for stage II progressive collapsing foot deformity: a finite element analysis

**DOI:** 10.1186/s13018-023-04216-3

**Published:** 2023-09-23

**Authors:** Fanglin Chen, Chengjie Yuan, Mian Liang, Guoping Le, Jian Xu

**Affiliations:** 1 Department of Orthopedics, Liuzhou Workers’ Hospital, Liuzhou, Guangxi China; 2 Department of Clinical Laboratory, Liuzhou Traditional Chinese Medical Hospital, The Third Affiliated Hospital of Guangxi University of Chinese Medicine, Liuzhou, Guangxi China; 3 Department of Orthopedics, The First Affiliated Hospital, Zhejiang University School of Medicine, Hangzhou, Zhejiang China

**Keywords:** Adult-acquired flatfoot deformity, Finite element model, Type II sinus tarsi implant, Foot biomechanical model

## Abstract

**Background:**

This study analyzed the advantages and disadvantages of different procedures for stage IIA progressive collapsing foot deformity (PCFD) through three-dimensional finite element models.

**Methods:**

A previous validated stage IIA PCFD FEA model was established consisting of 16 bones, 56 ligaments, 5 muscles and soft tissues. The ligament properties of the spring, deltoid, short plantar and long plantar ligaments, and plantar fascia were attenuated according to a previous publication. Medial column fusion (MCF), medializing calcaneal osteotomy (MCO), lateral column lengthening (LCL), and subtalar joint arthroereisis (SJA) operations were simulated in this model. The indexes of plantar stress distribution, maximum von Mises of the medial and lateral columns, strain of the medial ligaments and plantar fascia that supported the medial longitudinal arch, arch height, talo-first metatarsal angle, calcaneus pitch angle, and talonavicular coverage angle were all compared before and after simulated single-foot weight loading.

**Results:**

The maximum plantar stress of PCFD decreased with MCO and SJA but increased with MCF and LCL. MCF and LCL failed to significantly reduce the stress on the medial column fragments, thereby increasing their stress. Both MCO and SJA relieved medial plantar stress. MCF had no significant effect on stress relief of the medial ligament. MCO, LCL, and SJA were all shown to reduce the pressure on the medial plantar ligament, with LCL having the most obvious effect. All four procedures corrected the arch deformity; however, MCF was not as effective as the other methods. SJA is the best method for restoring arch height and correcting arch deformities. For stage IIA PCFD, isolated MCF failed to reduce pressure on the medial column; however, isolated MCO significantly reduced the pressure on the medial plantar and ligamentous soft tissues while restoring the foot’s arch and correcting the hindfoot valgus.

**Conclusion:**

SJA with type II sinus tarsi implant effectively transferred pressure from the medial plantar tract to the lateral side and restored the arch. Isolated LCL was not found suitable for stage IIA PCFD.

## Background

Progressive collapsing foot deformity (PCFD), previously termed adult-acquired flatfoot deformity (AAFD), is characterized by forefoot abduction, medial arch height loss, and hindfoot valgus. There are 4 main stages of deformity, in which stage II is regarded as passively correctable, flexible deformity of the forefoot and midfoot abduction and hindfoot valgus [1]. Stage II is divided into IIA and IIB, which reflects the extent of joint involvement and deformity. Stage IIA primarily involves the posterior tibial tendon dysfunction and hindfoot valgus, while Stage IIB includes significant joint deformity involving hindfoot valgus, forefoot abduction, and potential arthritis.

Previous studies have reported that common bony procedures such as naviculocuneiform (NC) fusion, or talonavicular (TN) fusion, medializing calcaneal osteotomy (MCO), and lateral column lengthening (LCL) achieved satisfactory clinical results in the treatment of stage II PCFD [2–5]. Osman et al. conducted a prospective randomized controlled study to compare the effect of LCL and MCO in the treatment of stage II PCFD and reported that both groups of patients showed significant improvements in function and imaging indicators compared with preoperative values [6]. Still, LCL had a better structural effect than MCO in correcting stage II PCFD and a lower complication rate. Gerrity et al. reported a series of patients with PCFD treated with MCF, all of which showed significant improvement in imaging parameters [7]. Ceccarini et al. reported that arthroereisis and tensioning of the posterior tibial tendon provided favorable functional outcomes in patients with stage II PCFD without arthritic manifestations under 60 years old [8].

Although those described surgical methods have shown good outcome for the treatment of stage II PCFD, there is currently no optimal treatment method available. Especially, few studies have compared the effect of different surgical methods on stage II PCFD through biomechanical analysis. In this study, we conducted various virtual surgeries using three-dimensional (3D) finite element models, performed biomechanical analysis, and compared the advantages and disadvantages of various surgeries for this stage deformity. We hypothesized that MCO and SJA could be helpful to shift the pressure on the medial plantar side to the lateral, and isolated LCL or MCF was not suitable for stage IIA PCFD from the perspective of biomechanical view.

## Methods

The volunteers approved and signed consent for this study. The study was approved by the ethics committee with an approval number of LW2021011.

### Reconstruction of 3-dimensional finite element stage II PCFD model

**Fig. 1:**
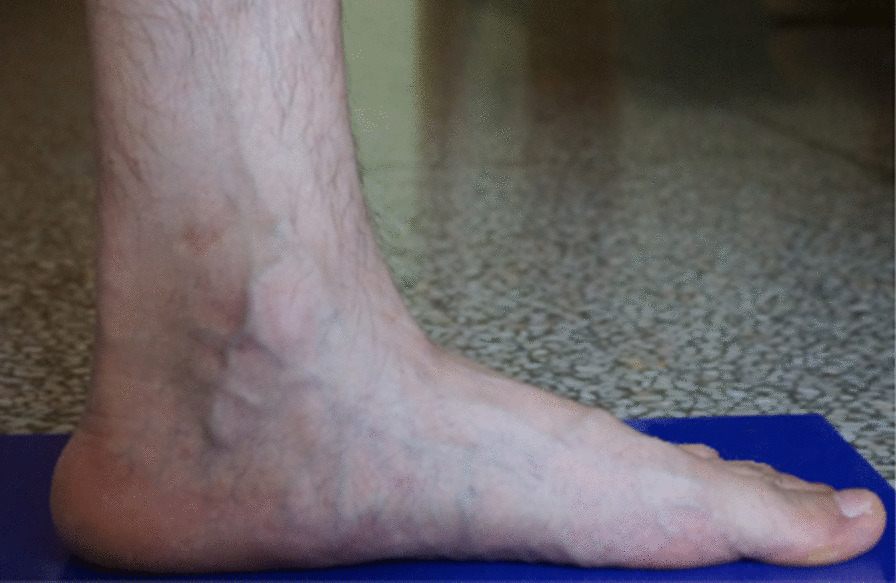
Gross appearance of a stage IIA flat foot volunteer

The current FE model was used from a previous validated model [9]. A 32-year-old male volunteer (height: 175 cm, weight: 60 kg) with stage IIA PCFD participated in the study (Fig. 1). The volunteer had no ankle fractures and foot and ankle tumors, which were verified by a radiographic examination under one-leg weight-bearing conditions. A computed tomography scan of the foot and ankle in a neutral position was also taken, and Digital Imaging and Communications in Medicine (DICOM) format data were collected.

The images were segmented in Mimics, version 17.0 (Materialise, Leuven, Belgium), to reconstruct the bone geometry that composes most of the foot and ankle: tibia, fibula, talus, calcaneus, cuboid, navicular, 3 cuneiforms, 5 metatarsals, and 2 sesamoids. The reverse engineering software Geomagic Studio, version 12.0 (Geomagic Inc., Research Triangle Park, NC) was used to reduce the noise levels of the STL format point cloud data, smooth the bone model surfaces, and generate nonuniform rational B-spline format models. Subsequently, the models were imported into the preprocessing finite element software HyperMesh, version 13.0 (Atlair Co., Troy, MI) to complete the reassembly and extraction of the cartilage models. The tendons and ligaments were established using truss units. The Achilles tendon and other five muscle tendons, namely tibialis posterior (TIBP), flexor hallucis longus (FHL), flexor digitorum longus (FDL), peroneus brevis (PB), and peroneus longus (PL), were incorporated into the model using bar elements, at their corresponding anatomical attachment sites. The finite element model of a flatfoot, consisting of 16 bones, 56 ligaments, and 5 muscles and soft tissues, was finally imported to the Abaqus software, version 6.12 (Abaqus Inc., Pawtucket, RI) for analyses (Fig. 1).

### Material properties

The properties (Young’s modulus and Poisson’s ratio) of the bone, soft tissue, and cartilage were assigned in accordance with published data [10, 11]. However, some of the ligament material properties in stage II PCFD, including the spring, deltoid, short plantar and long plantar ligaments, and plantar fascia, were not similar to those in the normal foot. Thus, these ligaments were assigned properties according to the reported data [12] (Table  1).

**Table 1:** Bone soft tissue and cartilage material parameter settings

Component	Elastic modulus *E* (MPa)	Poisson's ratio (*ν*)
Bone	7300	0.30
Soft tissue	1.15	0.49
Articular cartilage	10	0.49

### Loading and boundary settings

Two reference points were set just on the top of the tibia and fibula. The coupling relationship between the points and the upper end of the tibia and fibula was established. The ligaments were represented by 2-node truss units with non-compression characteristics that can only bear traction powers. The ligament functions were simulated with coupling units and changeable vector loads. Joint surface contacts were simulated with face-to-face nonlinear universal interaction. The surface contacts abided with tangential Coulomb friction (COF), and the friction coefficient was 0.1 [9]. Frictional contact interaction among the plantar surface and the ground was defined using a COF of 0.5 [13].

The midstance phase was simulated in these models. Vertical loads that were five-sixths and one-sixth of the body weight were applied to the two reference points to pass the load to the upper tibia and fibula surfaces [9]. The triceps surae, flexor hallucis longus tendon, peroneus longus muscle, peroneus brevis muscle, and flexor digitorum longus tendon in both models received reaction forces of 50%, 10.5%, 10%, 8.8%, and 6% of the corresponding body weight, respectively [12]. The analysis was performed after all the material properties, and boundary conditions were properly set up.

**Fig. 2:**
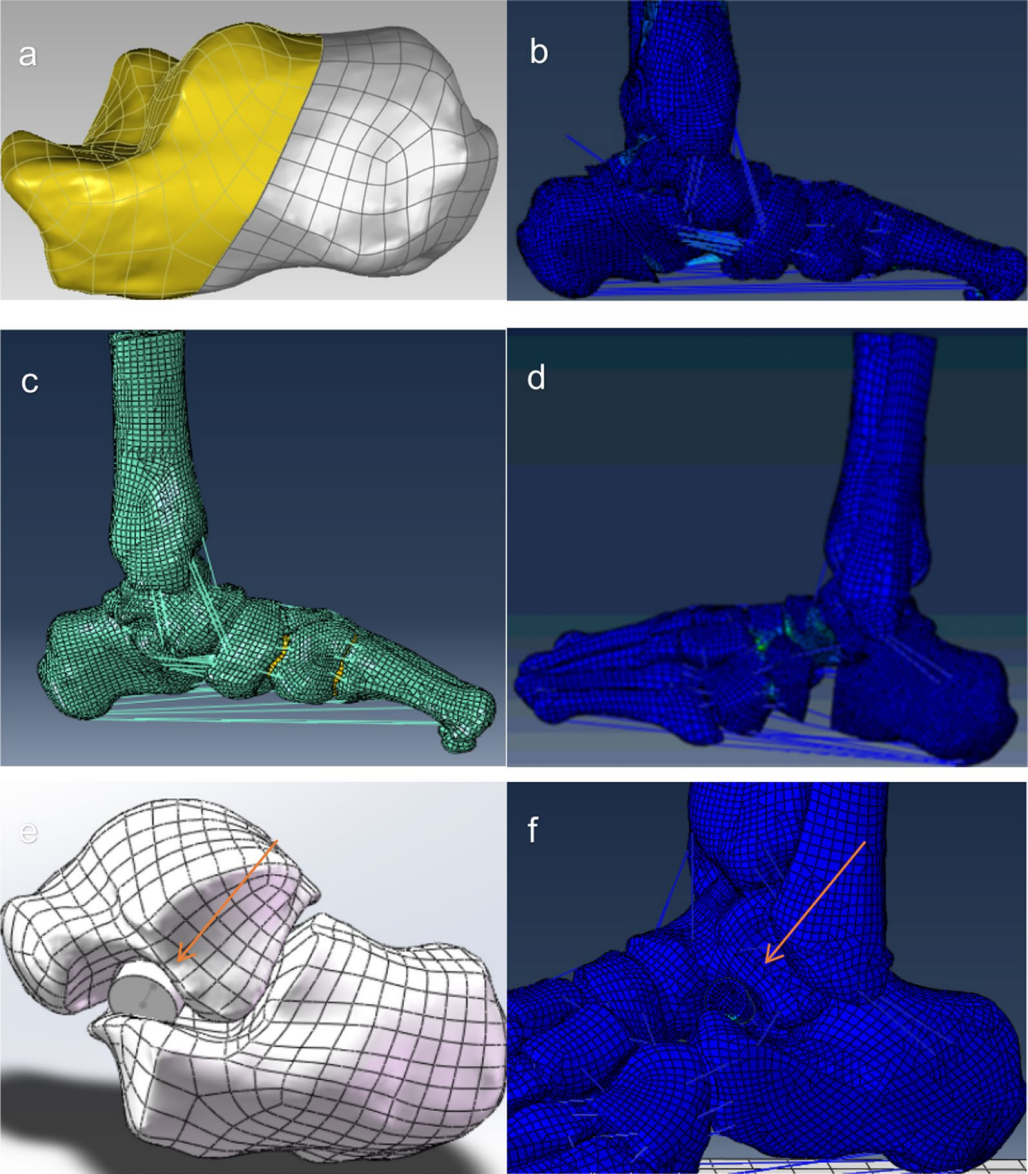
Simulate surgery based on 3D FEM (**a**–**f**). **a** and **b** Simulate MCO operation process. **c** Simulate MCF operation process, implant connecting bone and set relevant attributes. **d** Simulate the LCL operation process, and fill the space with the bone after simulation. **e** and **f** Simulate SJA operation process

Virtual surgeries were simulated as follows. MCF involved a fusion of the navicular and cuneiform bones, while MCO involved a 1 cm medialized osteotomy that extends from approximately 1–1.5 cm behind the posterior edge of the talus to the distal side of the calcaneus, at an angle of approximately 45°to the plantar plane of the foot. LCL refers to Evans osteotomy, which involves a transverse osteotomy approximately 10–15 mm away from the calcaneocuboid joint. The osteotomy site is then fixed with a 1-cm thick bone graft. SJA involves the implantation of an appropriate arthroereisis model, based on the size of the tarsal sinus tarsi, into the subtalar joint. The validity of the 3D FEM and virtual surgery simulation of MCF, MCO, LCL, and SJA were verified (Fig. 2).

In particular, the validity of the developed 3D FEM has been verified in previous publications by Xu et al. [9]. These preliminary models were established for different procedures using Geomagic, Solidwork, and Abaqus 6.14 (SIMULIA, USA) software.

### Evaluation index

The following parameters involving the distribution of maximal stress on the plantar surface, the maximum von-Mises values in the medial and lateral columns, the strain experienced by the medial ligaments and the plantar fascia supporting the medial longitudinal arch were evaluated, meanwhile, the height of the arch, the talo-first metatarsal angle, the angle of inclination of the calcaneus (calcaneus pitch angle), and the angle of coverage of the talonavicular joint (talonavicular coverage angle) were also assessed.

## Results

### Maximum plantar stress

**Fig. 3:**
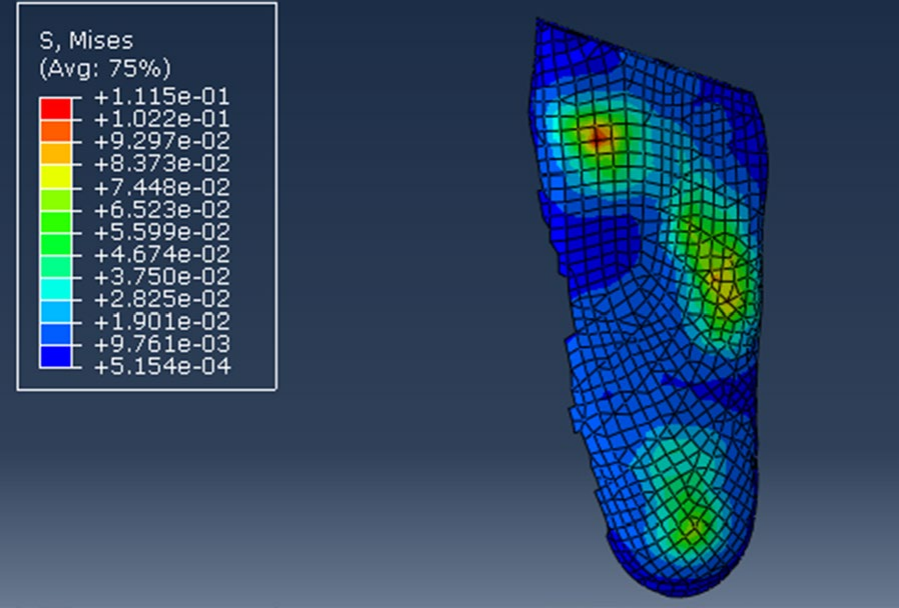
Simulation of the mid-stance phase in the mode, with the plantar stress distribution showing the peak stress of 111.5 kPA under the first metatarsal head

**Fig. 4:**
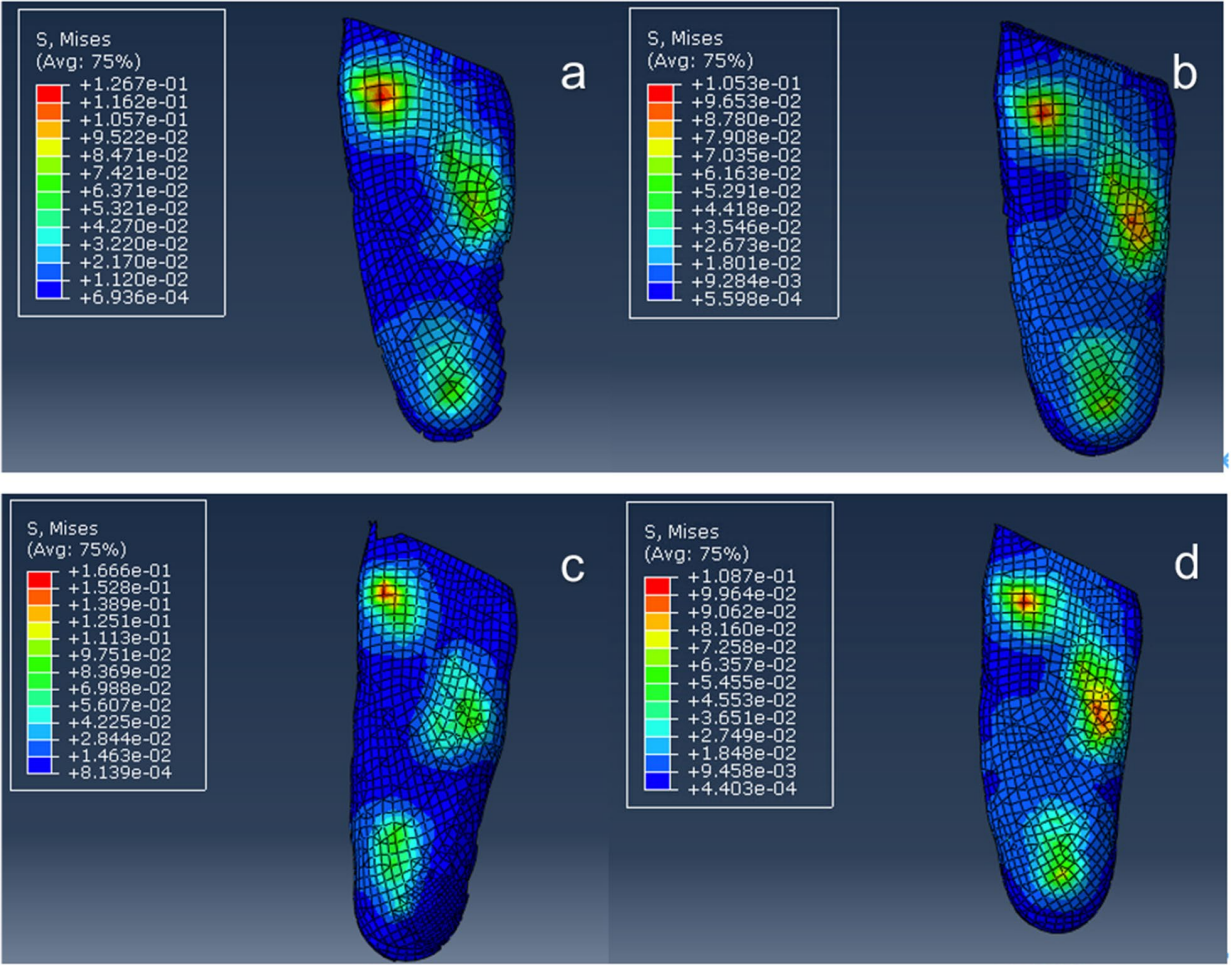
Changes in the maximum plantar stress. **a** The maximum plantar stress increased from 111.5 to 126.7 kPa after MCF. **b** The maximum plantar stress decreased to 105.3 kPa after MCO. **c** The maximum plantar stress increased to 166.6 kPa after LCL. **d** The maximum plantar stress and decreased to 108.7 kPa after SJA

Preoperatively, the midstance phase was simulated the plantar stress distribution displayed a maximum stress of 111.5 kPa under the first metatarsal head in the model (Fig. 3). Weight bearing was simulated after isolated MCF, MCO, LCL, or SJA, all of which had maximum plantar stress below the first metatarsal head. As shown in Fig. 4, the maximum plantar stress increased from 111.5 to 126.7 kPa after MCF, decreased to 105.3 kPa after MCO, increased to 166.6 kPa after LCL, and decreased to 108.7 kPa after SJA. In other words, the maximum plantar stress of PCFD decreased with MCO and SJA but increased with MCF and LCL.

### Bone stress change

The maximum von Mises stress values of the talus, navicular, medial cuneiform, first metatarsal, calcaneus, cuboid, and fifth metatarsal before and after the virtual procedures are listed in Table 2. MCF and LCL did not reduce the stress on the medial column fragments; instead, their stress increased. In contrast, both MCO and SJA relieved the stress on the medial column fragments.

**Table 2:** Comparison of maximum von Mises stress in the bones medial and lateral column of the finite element model under weight-bearing conditions after various virtual surgeries

Maximum von Mises stress in bones (MPa)	Weight-bearing				
	Preoperative	MCF	MCO	LCL	SJA
Medial column					
Talus	5.29	5.68	4.8	6	1.48
Navicular	4.4	3.97	3.97	5.1	3.87
Medial cuneiform	2.1	1.92	1.39	3.47	1.57
First metatarsal	3.2	3.4	2.2	10	2.5
Lateral column					
Calcaneus	4.15	3.97	4.2	9.7	1.5
Cuboid	2.58	2.19	2.59	4.8	2.57
Fifth metatarsal	3.5	3.5	9.73	6.24	9.9

*MCF* medial column fusion, *MCO* medializing calcaneal osteotomy, *LCL* lateral column lengthening, *SJA* subtalar joint arthroereisis

### Ligament and plantar fascia strain values

The maximum strain values of the anterior talofibular ligament (ATFL), posterior talofibular ligament (PTFL), tibiocalcaneal ligament (TCL), tibionavicular ligament (TNL), the spring ligament (SL), and plantar fascia (PF) are shown in Table 3. MCF had no significant effect on strain relief of the medial ligament. MCO, LCL, and SJA were all shown to reduce the pressure on the medial plantar ligament, with LCL having the most obvious effect.

**Table 3:** Comparison of the maximum strain in ligament and plantar fascia of the finite element model under weight-bearing conditions after various virtual surgeries

Maximum strain (MPa)	Weight-bearing				
	Preoperative	MCF	MCO	LCL	SJA
ATFL	0.93	0.61	0.98	0.69	0.84
PTFL	0.36	0.49	0.02	0.21	0.33
TCL	0.74	0.59	0.11	0.64	0.67
TNL	1.66	1.42	1.46	1	1.51
SL	3.48	4.39	3.13	1.87	2.6
PF	2	1.71	2.01	1	1.5

*MCF* medial column fusion, *MCO* medializing calcaneal osteotomy, *LCL* lateral column lengthening, *SJA* subtalar joint arthroereisis, *ATFL* anterior talofibular ligament, *PTFL* posterior talofibular ligament, *TCL* tibiocalcaneal ligament, *TNL* tibionavicular ligament, *SL* spring ligament, *PF* plantar fascia

### Radiographic parameter measurement

Table 4 shows the arch height, talo-first metatarsal angle, calcaneus pitch angle, and talonavicular coverage angle before and after virtual surgery. After the MCF simulation, the talo-first metatarsal angle, calcaneus pitch angle, and talonavicular coverage angle changed from 8.3°, 14.4°, and 11.7° to 5.2°, 15.6°, and 8.2°, respectively. Arch height was restored from 10 to 15 mm. After the MCO simulation, the corresponding angles were 7.3°, 16.1°, and 10.1°, while the arch height was restored to 18 mm. After the LCL simulation, the corresponding angles were 5.1°, 17.1°, and 7.6°, and arch height was restored to 18 mm. After the SJA simulation, the corresponding angles were 4.2°, 16.9°, and 4.3°, and arch height was restored to 19 mm. In other words, all four procedures corrected the arch deformity. Although MCF corrected the deformity, it was not as effective as the other methods. SJA is the best method for restoring arch height and correcting arch deformities.

**Table 4:** Comparison of the measured radiographic parameter in the finite element model under weight-bearing conditions after various virtual surgeries

Evaluation index	Weight-bearing				
	﻿Preoperative	MCF	MCO	LCL	SJA
Arch height (mm)	10.0	15.0	18.0	18.0	19.0
Talo-first metatarsal angle (°)	8.3	5.2	7.3	5.1	4.2
Calcaneus pitch angle (°)	14.41	15.6	16.1	17.1	16.9
Talonavicular coverage angle (°)	11.78	8.2	10.1	7.6	4.3

*MCF* medial column fusion, *MCO* medializing calcaneal osteotomy, *LCL* lateral column lengthening, *SJA* subtalar joint arthroereisis

## Discussion

In stage II PCFD, the medial column is unstable, which is one of the important causes of the arch collapse. Various treatment modalities are currently used to stabilize the medial plantar ray and treat valgus deformities of the hindfoot. It is common to treat stage II PCFD using surgical modalities, such as osteotomy, and tendon transposition or reconstruction, either alone or in combination with surgery. Although fusion surgery can correct the deformity to a considerable extent, the motion between the fused joints is lost, adjacent joints are stressed, and the normal physiological movement mechanism of the foot is correspondingly changed. Roling et al. [14] showed that the navicular cuneiform joint has the highest mobility in the medial column, accounting for approximately 50% of the sagittal plane activity of the first array.

Although medial column stabilization could correct varus deformity of the forefoot and stabilize the midfoot, our study shows that MCF only reduces the maximum stress on the corresponding fusion joints. Further, MCF fails to reduce the pressure on the plantar soft tissue and even increases the maximum stress on the soles. This is especially evident in the stage IIA PCFD model following MCF, where the maximum stress on the plantar surface increased by 13.6%. This study also showed that the maximum strain of the spring ligament after MCF is higher than its baseline value before surgery. The reason might be related to the instability of the TN joint and the sinking of the talus head, which causes an increase in the force over the spring ligament. According to the parameters we measured at various angles, MCF can partially restore the medial arch, thus preventing the talus head from sinking while weight-bearing. However, despite some effects in correcting deformities, the effect is not significant. Thus, we conclude that isolated medial column stabilization surgery cannot help patients with stage IIA PCFD relieve the stress on the medial column; however, this surgery can aggravate the pressure on the adjacent joints and plantar soles.

However, MCO was shown to reduce the pressure of the inner arch, normalize the force of the talonavicular joint, and relieve the symptoms of PCFD, which is considered an effective way to correct the valgus deformity of the foot after stage II PCFD [15–18]. MCO mediates the insertion of the Achilles tendon relative to the subtalar joint axis, thereby increasing the calcaneus varus moment and limiting the degree of hindfoot valgus before forefoot weight-bearing [19, 20]. The mechanism by which MCO improves the linkage between the hindfoot joints is not completely clear, but many clinical studies have reported satisfactory results in improving deformities and symptoms. Myerson et al. [21] described 129 patients with PCFD who were treated with MCO and flexor digitorum longus transposition, with good results. The talo-first metatarsal and talonavicular coverage angles in these patients were significantly corrected after surgery. Otis et al. [22] measured the length of the spring ligament of the specimen foot before and after the MCO under load, and confirmed that the MCO can effectively reduce the tension of the spring ligament and help stabilize the medial structure. According to our findings, for stage IIA PCFD, even without soft tissue or other osseous surgeries, MCO could significantly reduce the pressure on the medial metatarsal bone fragments while increasing the stress of each bone fragment of the lateral column, especially in the fifth metatarsal and cuboid. MCO significantly reduces the strain on the medial ligament and spring ligament, and it also has a significant effect on the restoration of the arch of the foot and correction of the valgus of the hindfoot, which has also been confirmed in many clinical studies.

We found that the plantar stress under the first metatarsal head in stage IIA PCFD increased significantly after LCL, from the preoperative 111.5–166.6 kPa. The maximum stress of each bone fragment of the medial column increased, with the maximum stress of the first metatarsal more than tripling. Although LCL can significantly reduce the maximum strain of the spring ligament, medial ligament, and plantar fascia and has a same effect of MCO on arch restoration and better deformity correction, the mechanism for this is not ideal. LCL makes the normal forefoot alignment more adducted and increases compressive stress after the medial column is squeezed, thereby reducing the strain on the medial ligament spring ligament. LCL affects the alignment of the foot and increases stress on the medial column; therefore, we believe that LCL is unsuitable for stage IIA PCFD. This is consistent with the fact that many scholars believe that good forefoot alignment is a contraindication for this surgery, and we have confirmed this from a biomechanical point of view.

In this study, we performed SJA using a type II sinus tarsi implant. Studies [9, 23] have shown that SJA can effectively reduce pressure on the medial longitudinal arch and transfer pressure to the lateral side. In addition, it can reduce the tension on the tibial nerve, plantar fascia, and posterior tibial tendon. According to this study, SJA can significantly reduce the stress on each bone fragment of the sole and medial column and transfer the medial stress to the lateral side, thereby increasing the stress on the lateral column bone fragments. At the same time, it significantly reduces the strain on the medial and spring ligaments. Wong et al. [23] also confirmed that the change in medial stress helps prevent the deterioration of PCFD and believed that SJA could reduce medial load and thus effectively prevent arch collapse. We compared the relevant parameters after MCO with those after SJA and found that the maximum stress value of the medial column bone fragment after the type II sinus tarsi implant was equivalent to the effect after MCO. SJA positively reduces the compressive strain of the spring ligament and plantar fascia, and its effect is more pronounced than that of MCO. At the same time, the type II sinus tarsi implant also has better results in correcting deformities, and its correction effect on hindfoot valgus is comparable to that of MCO. This is because of its distracting effect on the sinus tarsi, which elevates the talar head and navicular height. Therefore, SJA is more effective than isolated MCO in arch restoration and deformity correction.

### Limitations

This study only analyzed the effect of different surgeries on stage IIA PCFD by establishing a 3D finite element model. Although the model established in this study is representative to a certain extent, the number of cases is limited, and there are individual differences in disease and deformity among different patients, which may compromise the validity of the results. In addition, there is a significant difference in elastic modulus between cortical and trabecular bones (14,000 vs. 350 MPa). Assigning the same material properties to these bones has a negative impact on the outcomes when it comes to evaluating the stress distribution. For subsequent research, a heterogeneous bony configuration based on CT Hounsfield values would be suggested.

## Conclusion

For stage IIA PCFD, isolated MCF failed to reduce pressure on the medial column, while isolated MCO significantly reduced the pressure on the medial plantar and ligamentous soft tissues while restoring the foot arch and correcting the hindfoot valgus. SJA with type II sinus tarsi implant effectively transferred pressure from the medial plantar tract to the lateral side and restored the arch. Its pressure-reducing effect was comparable to that of MCO, but its deformity-correcting effect is better than that of the latter. Isolated LCL is unsuitable for stage IIA PCFD as it not only increases the adduction of the forefoot but also increases the maximum stress on the medial plantar.

## Data Availability

The datasets generated and analyzed during the current study are not publicly available due to the large size of finite element model but are available from the corresponding author on reasonable request.

## Abbreviations

PCFD: Progressive collapsing foot deformity
MCF: Medial column fusion
MCO: Medializing calcaneal osteotomy
LCL: Lateral column lengthening
SJA: Subtalar joint arthroereisis
AAFD: Adult-acquired flatfoot deformity
NC: Naviculocuniform
TN: Talonavicular
3D: Three-dimensional
DICOM: Digital imaging and communications in medicine
TIBP: Tibialis posterior
FHL: Flexor hallucis longus
FDL: Flexor digitorum longus
PB: Peroneus brevis
PL: Peroneus longus
COF: Coulomb friction
ATFL: Anterior talofibular ligament
PTFL: Posterior talofibular ligament
TCL: Tibiocalcaneal ligament
TNL: Tibionavicular ligament
SL: Spring ligament
PF: Plantar fascia
